# A review of *Legionella* transmission risk in built environments: sources, regulations, sampling, and detection

**DOI:** 10.3389/fpubh.2024.1415157

**Published:** 2024-07-26

**Authors:** Xiao Hui Yao, Fan Shen, Jing Hao, Lu Huang, Bin Keng

**Affiliations:** ^1^Department of Environmental Health, Beijing Center for Disease Prevention and Control, Beijing, China; ^2^Department of Environmental Health, Beijing Fengtai District Center for Disease Prevention and Control, Beijing, China; ^3^Department of Environmental Health, Beijing Dongcheng District Center for Disease Prevention and Control, Beijing, China; ^4^Department of Environmental Health, Beijing Huairou District Center for Disease Prevention and Control, Beijing, China

**Keywords:** *Legionella*, built environment, aerosol, transmission risk, monitoring

## Abstract

The risk of *Legionella* transmission in built environments remains a significant concern. *Legionella* can spread within buildings through aerosol transmission, prompting the exploration of airborne transmission pathways and proposing corresponding prevention and control measures based on building characteristics. To this end, a comprehensive literature review on the transmission risk of *Legionella* in built environments was performed. Four electronic databases (PubMed, Web of Science, Google Scholar, and CNKI) were searched from inception to March 2024 for publications reporting the risk of *Legionella* transmission in built environments. Relevant articles and gray literature reports were hand-searched, and 96 studies were finally included. *Legionella* pollution comes from various sources, mainly originates in a variety of built environments in which human beings remain for extended periods. The sources, outbreaks, national standards, regulations, and monitoring techniques for *Legionella* in buildings are reviewed, in addition to increases in *Legionella* transmission risk due to poor maintenance of water systems and long-distance transmission events caused by aerosol characteristics. Air and water sampling using various analytical methods helps identify *Legionella* in the environment, recognize sources in the built environments, and control outbreaks. By comparing the standard regulations of national organizations globally, the authors further highlight gaps and deficiencies in *Legionella* surveillance in China. Such advancements offer essential insights and references for understanding and addressing *Legionella* transmission risk in the built environment, with the potential to contribute to safeguarding public health and building environment safety.

## 1 Introduction

*Legionella* is a pathogenic gram-negative bacterium and the pathogen associated with Legionellosis. Legionellosis represents two types of illnesses: one is a mild flue like illness known as Pontiac fever (1), and the second is a fatal pneumonia known as Legionnaires' disease, with a mortality rate between 3 and 33% (2, 3). *Legionella* can affect anyone but it principally affects individuals who are susceptible due to age, illness, immunosuppression, or other risk factors, such as smoking (3). From 2000 to 2018, the reported incidence of Legionnaires' disease increased more than 6-fold. Risk environments and susceptible populations are found globally, but most currently available epidemiologic data focus on Legionellosis in large metropolitan areas in developed regions. The problem of *Legionella* is potentially underappreciated and underestimated by a factor of eight to ten (3). The spread of *Legionella* is mainly related to the aerosol atomization of bacteria from water to air. Therefore, the transmission risk is higher in built environments with aerosol production characteristics (4, 5). The built environment mainly refers to residential, multi-purpose commercial, and mixed-use buildings, including apartments, offices, hotels, hospitals, and schools (6). A variety of familiar water sources in the built environment, including showers, faucets, hot tubs, swimming pools, cooling towers, and fountains, can produce large amounts of aerosols from heated water sources contaminated with *Legionella* that can spread over long distances (7). Although many sources are associated with Legionnaires' disease, water systems often have conditions conducive for *Legionella* growth, including suitable water temperature, water retention, the presence of scale, sediments, and biofilms in pipes and fixtures, and the absence of disinfectants (8). A common route of transmission is inhaling water droplets or aerosols in a contaminated environment, resulting in infection with Legionnaires' disease. Because humans are accidental hosts of *Legionella*, Legionnaires' disease is generally not considered to spread from person to person.

Despite increased global awareness of Legionnaires' disease and efforts to control *Legionella* spread, outbreaks continue to occur, the sources of pathogens are expanding, and the number of reported cases shows no sign of decreasing (9). In 2023, Poland experienced an outbreak of Legionnaires' disease, with a total of 166 confirmed cases and 23 deaths, resulting in a mortality rate of 14% (10). Due to its high morbidity and mortality, Legionnaires' disease has become a significant public health concern. Epidemiological data show that most reported *Legionella* cases occur in large built environments (3), and tourism-related Legionnaires' disease outbreaks may occur in cruise ships, hotels, or resorts. A common feature of such environments is the existence of a large complex water supply system and lack of adequate maintenance (11). *Legionella* is ubiquitous in freshwater resources globally; however, the number of *Legionella* in such environments is insufficient to cause the disease, and the conditions for the proliferation of *Legionella* are not as conducive as those in the built environment (11). Therefore, the spread of *Legionella* in built environments requires attention due to its significance. We should implement intervention measures to reduce the spread of *Legionella* and prevent outbreaks of *Legionella* infections.

Legionnaires' disease occurs worldwide. The incidence of Legionellosis varies greatly based on the level of surveillance and reporting across countries. The lack of robust surveillance systems may lead to underreporting and poor control measures for *Legionella in* developing countries, posing a significant public health challenge. The current status of *Legionella* management in China, as in developing countries such as India, is an area that requires further attention and research, as well as robust surveillance systems, guidelines, and regulations (12). This review focuses on research related to the risk of *Legionella* transmission in the built environments, which could improve building safety, facilitate development of prevention and control strategies, enhance surveillance capacity, and facilitate the effective management of *Legionella* infection.

## 2 Materials and methods

A comprehensive search of the academic databases PubMed, Web of Science, Google Scholar, and CNKI was conducted. Specific terms, such as “*Legionella* and built environment,” “*Legionella* and aerosols,” “*Legionella* and transmission risk,” “*Legionella* aerosol and sampling,” and “*Legionella* aerosol and detection” were searched. The titles and abstracts of the articles were screened and assessed for inclusion in the review. Articles were cross-checked, and duplicated and irrelevant articles were excluded. A list of 747 papers was discussed extensively to resolve any discrepancies and reach consensus. Finally, 96 references were included in the present, out of which 79% were published from 2010 onward.

## 3 Built environment and Legionnaires' disease

In recent years, with an increase in urbanization, equipment for aerosol production has been introduced into buildings in large numbers. Improper design and poor operational management have resulted in areas becoming breeding grounds and dissemination sites for *Legionella*. Therefore, the risk of outbreaks and epidemics is increasing gradually.

### 3.1 Cooling towers

Cooling towers are essential components of centralized air-conditioning systems. Their structures and operating temperatures are highly suitable for the growth and reproduction of *Legionella*, making them crucial sources for Legionnaires' disease outbreaks in public buildings. If cooling towers are not cleaned and disinfected promptly, protozoa multiply, providing conditions for the growth of *Legionella*. A study conducted in 44 hospitalized medical institutions in Spain from 2005 to 2006 found that *Legionella* accounted for nearly 24% of the 470 cooling tower samples collected (13). Similarly, testing 96 cooling towers in public buildings in Greece showed that almost 49% of those towers were infested by *Legionella* (14).

### 3.2 Water supply system

Building water supply systems, including hot water showers, hot springs, and swimming pools, are common in modern cities, and is an ideal breeding environment for *Legionella*. Large buildings with centralized hot water systems often have complex pipelines, in which certain conditions, including low residual disinfectants, favorable water temperature, high water age, sediment accumulation, and free-living amoebae, support the growth of bacteria in biofilms. *Legionella* is often found in stagnant or unused pipe sections, intermittently used water tanks, and other places, which can amplify ideal living environments (15). The optimal growth temperature for *Legionella* is between 25 and 45°C, which is common in hot water pipes of building water supply systems. Samples of hot water systems in 40 hotels in five different regions of Italy revealed the presence of *Legionella* in 75% of the buildings (16). Changes in water flow or flushing can disrupt biofilms and release *Legionella* downstream (17). A research report stated that compared with that in high-usage faucets, the number of *Legionella pneumophila* in low-usage faucets was increased 125 times, with more stagnant water and more significant water flow variations (18). Hospital-acquired Legionnaires' disease is also associated with *Legionella* in water-supply systems (19). A recent study in Hungary found *Legionella* in hot water systems of 92% of the investigated healthcare facilities (*N* = 24), with a median exceeding 1,000 CFU/L in seven hospitals (20).

### 3.3 Medical equipment

The occurrence of Legionnaires' disease in hospitals that use tap water is related to aerosol-generating equipment (21). Hamilton et al. (22) found that the surface-area-to-volume ratio of water pipes in dental clinics is high, which is conducive to biofilm growth. Combined with high-pressure water equipment, fine aerosols can be generated in the breathing zone, posing a potential risk of *L. pneumophila* inhalation. Built environment exposure to aerosol-producing water fixing devices, including medical and dental equipment, will atomize water containing *Legionella* into easily-inhaled aerosols during work (23, 24). These devices are an essential part of modern urban building environments, in which conditions are conducive to the growth and proliferation of *Legionella*, posing a high risk of transmission.

### 3.4 Green building

At present, green building is promoted vigorously, and its essential goal is the conservation of energy, water, and materials. Water-saving characteristics are due to the need to reduce unsustainable water intake, especially in response to drought and other consequences of climate change. Energy conservation involves reducing dependence on fossil fuels and limiting greenhouse gas emissions; however, such measures often also have effects on water supply systems and increase *Legionella* breeding risk in building water supply systems (25).

The extremes of green buildings are off-grid or “net zero” buildings that do not rely on external water networks to provide drinking water or wastewater services (26). The primary goal of certification, such as the “Living Building Challenge,” is for this building to be independent of water facilities. It is characterized by using water-saving equipment to reduce water consumption, in addition to rainwater collection, reservoirs, etc. However, such design configurations pose unique challenges and corresponding public health problems. Leadership in Energy and Environmental Design (LEED) certified green buildings typically save 20–50% of drinking water. Water-saving measures inherently increase stagnation and overall water age, both at the municipal level (i.e., primary water distribution system) and at the building level (i.e., water age of house pipes) (27). One survey found that in a typical LEED-certified healthcare suite, the water age was 8 days old, while in an off-grid office suite, it was more than 6 months old (27). In addition, excessively high water age can also aggravate corrosion, taste, and odor problems and cause loss of disinfectant residues and regrowth of microorganisms, damaging to the primary water distribution systems (28). In a previous study, gene copies of *Legionella* were not detectable in the incoming water supply in LEED-certified healthcare suites. Still, they ranged between 10,000 and 100,000 GC/mL in three out the five housing pipeline sampling locations (27).

Green buildings can also save energy by lowering the temperature and creating conditions conducive to *Legionella* growth (29). Lowering the temperature of water heater outlets could increase the possibility of *Legionella* detection and the degree of contamination of faucets significantly. Moreover, turning off the recirculation system at night causes stagnation for up to 8 h or longer. Even in thermal insulation systems, water reaches the desired temperature for *Legionella* growth during such a long arrest period (30). Darelid et al. conducted a 10-year *Legionella* surveillance program; according to the results, maintaining the circulating hot water temperature > 55°C could limit bacterial growth and thus control the disease outbreak (31). As part of the water management plan, green buildings should pay close attention to maintenance of the temperature of hot water at 55–60°C and cold water at <25°C (3). In addition, use of heat pumps or solar water heaters may lead to water temperatures beneficial for *Legionella* growth. Rhoads et al. observed that the added storage incurred by a solar water heater in a net zero energy home increased the hot-water age from <1 day to between 2 and 3 days (27). Moreover, the solar preheated tank may be within the optimal temperature range for *Legionella* growth on cloudy days. The copy number measured by qPCR in the hot water manifold receiving hot water and delivering it to the faucet was significantly higher (>106 GC/mL) (27).

### 3.5 Transmission risk

The literature indicates that aerosol transmission can occur over a wide range, from a few to several thousands of meters (32, 33), with records showing that aerosols containing *L. pneumophila* are capable of infecting persons residing at a distance of >6 km from the contaminated source (34).

Most outbreaks are associated with healthcare and hotel buildings, as well as buildings supplied by large (>10,000 people) water systems (35). In 1994, 50 passengers on a cruise ship were infected with *Legionella* due to exposure to contaminated hot springs. Research has found that for every additional hour spent in hot springs, the risk of contracting Legionnaires' disease increases by 64% (36). In 2005, the lack of proper maintenance of a decorative fountain in a restaurant in South Dakota led to 18 people being infected with *Legionella* (37). In contrast to retrospective investigations of *Legionella* outbreaks abroad, domestic studies in China have mainly focused on the current status of *Legionella* contamination in public environments. For example, Zhang et al. (38) studied the hot water supply system of a hotel building in East China and reported an *L. pneumophila* detection rate of 50%. The highest contamination of *L. pneumophila* was found in the heat exchanger, which was attributed to the low hot-water temperature and high turbidity of the hotel. Zhang et al. (39) investigated *L. pneumophila* contamination in the centralized air-conditioned cooling water of public transport buildings, with an average detection rate of 47.70%. Therefore, the risk of *Legionella* transmission in building environments is high. It is vital to monitor and manage *Legionella* incidents and improve Legionnaires' disease risk alerts and prevention measures.

## 4 Major *Legionella* outbreaks

An outbreak of *Legionella* is usually associated with buildings or structures with complex water supply systems, such as hotels, long-term care facilities, hospitals, and cruise ships. Since its discovery, Legionnaires' disease has occurred in several countries, including the United States (40), Sweden (41), Netherlands (42), Italy (43, 44), Spain (7, 45), and Japan (46). China has also reported *Legionella* detection multiple times, with numerous outbreaks of Legionnaires' disease in Beijing alone (47, 48). In comparison, areas with relatively high medical standards have a better understanding and detection of *Legionella*. Modern equipment, such as fountains, showers, and air conditioning, are more commonly used in these areas, increasing the likelihood of *Legionella* infection. Outbreaks often occur in developed cities. The specific events are shown in Table 1.

**Table 1:** Typical events of *Legionella* outbreak in built environment water systems in different countries from 1976 to 2018: sources, process and characteristics.

**Location**	**Time**	**Scene**	**Source**	**Event process**	**Characteristic**	**References**
Philadelphia, USA	August 1976	Hotel	Cooling tower	An outbreak of pneumonia occurred at the Veterans Convention held at the Philadelphia Hotel in the United States in August 1976. Exposure may have happened in the lobby of the headquarters hotel or in the area immediately surrounding the hotel. It was eventually found that this pneumonia was due to bacterial-containing aerosols formed by the operation of the cooling towers entering the central air-conditioning system. There were a total of 182 cases in the outbreak, and 29 infected people died.	Premiere case	(40)
Sweden	December 1990	Hospital	Hot water system	An outbreak of Legionnaires' disease occurred at the general hospital in Varnamo, Sweden, where 28 patients and three staff members developed pneumonia, and three died, all of whom had been in contact with the hospital's hot water between 2 and 11 days before the onset of the disease. During the outbreak, the water temperature in the hospital wards was 43°C, which was suitable for the growth and reproduction of *Legionella*. *Legionella* was tested and isolated from hot water supplied to 17 of 20 hospital wards, probably spread by misting of shower nozzles, and an increase in the temperature of the hot water from 45 to 65°C, together with thermal disinfection of the showers, contained the outbreak within 1 week.	Case of Legionnaires' disease outbreak due to low hot water temperature in hospital	(41)
Beijing, China	June 1997	Office building	Air Conditioning Condensate	In Beijing, An outbreak of upper respiratory tract infection occurred among employees in an office building. This office building is a newly completed building with a fully enclosed design and poor ventilation. An investigation into the air conditioning system of the building revealed that there are ~30 fan coil units in the ceiling on each floor of the building. The fresh air transmitted from the central air conditioning to each floor is blown through the fan coil units and cooled by chilled water coils before entering each room. During the process, the condensate in the condensate tray was blown into the office with fresh air in the form of aerosols, causing infection of people and resulting in 108 patients falling ill.	A case of air-conditioning system causing mixed infections of *L. pneumophila* multocida in a collective unit population	(47)
Netherlands	February 1999	Flower show	Spas	During the outbreak of Legionnaires' disease at the Netherlands Flower Show in February 1999, when 188 participants were sickened, an environmental survey of the exhibition halls revealed that the disinfection system in hall 3, which was the most excellent source of transmission, had been in operation for the duration of the show and had never been used before, and that the disinfection system had failed. Two spas in halls 3 and 4, and a sprinkler in hall 8, were culture-positive for *L. pneumophila*.	Large outbreaks second only to first cases, while more than 16% of cases had an incubation period of more than 10 days	(42)
Beijing, China	January 2000	Boot camp	Hot water for the shower	An outbreak of legionellosis caused by *Legionella* Bozeman occurred in a recruit training camp in the suburbs of Beijing, with 18 patients out of 203 recruits, including two cases of pneumonia and 16 cases of Pontiac fever. The patients all lived in new buildings, which were opened too early, and the indoor humidity and the winter heating season made it easy to generate aerosols. Moreover, the newly built bathrooms were poorly ventilated initially.	The first case of *Legionella* Bozeman in China	(48)
Beijing, China	August 2003	Plant	Showerhead	An outbreak of non-pneumonic Legionnaires' disease-Pontiac fever caused by contamination of the showerheads in the hot water shower system occurred among the employees of a factory in Beijing, with 81 cases of centralized respiratory fever occurring within a period, representing an incidence rate of 14.86%. The shower system of the communal bathroom was not in use for an extended period before the production in July, which led to the considerable accumulation of *Legionella* bacteria, and it was not replaced and disinfected after entering the centralized production in the middle of July. The showerheads were replaced and disinfected.	Domestic Pontiac fever case	(49)
Italy	2004	Hospital	Water pipes	An epidemic cluster of infections caused by *Legionella pneumophila* serogroup 1 occurred in a hospital in northern Italy, involving six infections and two deaths. The hospital's water supply system had been disinfected several times since 1990, and the installation of continuous chlorine dioxide generators did not significantly reduce contamination with *Legionella* species. The lack of effectiveness of the disinfection treatments may have been due to hospital-specific conditions or the structural complexity of the plumbing system. Molecular typing linked the outbreak to the contamination of the hospital's water system and demonstrated the persistence of a predominant strain of *L. pneumophila* for 15 years.	A case of prolonged presence of the same *Legionella* strain in a hospital water supply system	(43)
Spain	October 2005	Communal	Cooling tower	An outbreak of 55 infections with a 5.5% case-fatality rate was reported in the Catalan Legion in Spain. The source was the cooling towers of plant A in Gurb, where high temperatures, high humidity, and virtually no wind led to widespread horizontal airborne spread of contaminated aerosols, with a wide distribution of cases, including six cases 2.5–3.4 km outside the source of infection.	Aerosol long-range transport case	(7)
Japan	June 2015	Spa center	Spa	An outbreak of Legionnaires' disease in a spa center in Odawara City, Kanagawa Prefecture, Japan, infected seven patients. Five of the seven patients were regular visitors to the spa center, and some revisited the spa after the onset of the disease. Epidemiological investigations and laboratory results indicated that failure to chlorinate the bathing water and the circulatory system adequately resulted in *Legionella* colonization of the spa.	Case caused by *L. pneumophila* serotypes 1 and 13 together	(46)
Spain	November 2017	Communal	Hot tub	An outbreak of 27 cases of Legionnaires' disease, including one fatality, has been reported in the Palma Nova region of Spain. All cases were travelers staying in ten hotels or tourist flats in the affected area. In one of the cases, working in a hotel not previously associated with the cluster, the investigation identified a private hot tub on the terrace of a hotel penthouse flat, a vacant room with a hot tub filled with water, but a lack regular inspections and maintenance, coupled with the location on the rooftop and wind dispersal, which led to the spread of aerosols, and the collection of 48 water samples, of which, 38 samples were positive for *Legionella pneumophila* serotype 1.	Case of weather-facilitating aerosol propagation	(45)
Italy	July 2018	Communal	Public fountain	A case-control study and aerosol dispersal investigation were carried out in a significant legionellosis outbreak in Bresso, Italy, where fountains were the cause of the epidemic. Extreme rainstorms occurring 5–6 days before the outbreak increased the risk of infection, and *Legionella* bioaerosols emitted from the fountains may have entered surrounding buildings. Outdoor bioaerosols have a high penetration efficiency. Fifty-two cases were diagnosed in this outbreak, including five deaths.	Case of increased risk of aerosol transmission from stormy weather	(44)

Outbreaks have continued since *Legionella* was first detected, resulting in multiple infections and even death and posing a severe threat to public health and safety. These incidents have aroused public concern about *Legionella* infections and heightened awareness and vigilance against Legionnaires' disease, prompting the government and relevant departments to introduce stricter regulations and standards to regulate the management of cooling towers and water systems in public places and to strengthen monitoring and preventive and control measures to reduce the likelihood of recurrence of similar incidents. Aerosols play an important role in significant events and can spread over long distances under the influence of weather. At the same time, in areas with well-established routine surveillance systems, it is easy to detect trends in legionellosis outbreaks at an early stage and trace the source of environmental contamination, so that effective measures can be taken to control the further evolution of legionellosis outbreaks.

## 5 *Legionella* monitoring regulations

Incidents of *Legionella* morbidity in buildings occur nationwide, and the risk of the disease and difficulty in treating it is of great concern to Governments and the World Health Organization (WHO). Around the world, regulatory agencies and health organizations have issued and developed recommendations and regulations aimed at controlling and preventing the risk of contracting Legionellosis, including the sanitation of water supplies, maintenance through regular cleaning, and disinfection of facilities and equipment to reduce the presence of the pathogen.

### 5.1 World Health Organization

In 1987, the WHO issued guidelines for controlling *Legionella* in water sources and supply systems, emphasizing the management of water quality and supply facilities to reduce the risk of *Legionella* infections. In 1993, the WHO formally declared *Legionella* infection a global problem, advocating for broader guidance on prevention, diagnosis, and treatment. In 2007, the WHO published “*Legionella* and the prevention of legionellosis,” guiding the assessment and management of risks associated with potential hazardous environments such as cooling towers, swimming pools, and hot springs. The document also identifies necessary measures to prevent or adequately control *Legionella* exposure risk in specific environments (50). In 2009, the WHO issued the WHO guidelines for indoor air quality: dampness and mold, which guides public health and other authorities in planning or formulating regulations, action plans, and policies to increase safety and ensure healthy building conditions (51). In 2017, the WHO made suggestions regarding water system construction, design, routine operational monitoring, and management incorporation in water safety plans developed by building owners or managers (52). The WHO recommends implementing water safety plans, based on Hazard Analysis and Critical Control Points (HACCP) principles, to proactively identify, assess, and manage risks related to water quality. This can be an effective method for actively managing *Legionella* contamination risks in various environments such as hospitals, long-term care facilities, spas, and hotels. Continuous research and implementation of effective control measures are essential for ensuring water safety and minimization of the spread of *Legionella* in water systems. For example, Salah et al. (53) discussed the successfully implementation of a water safety plan at UAB Medical Center, highlighting the importance of water system management plans in guiding risk assessment and mitigation strategies. Marino et al. (54) also highlighted the importance of comprehensive water safety plans in dental clinic complexes in identifying new risk factors associated with *Legionella* and *Pseudomonas aeruginosa* contamination.

### 5.2 European Union

*Legionella*-related research in Europe has been continuing for a significant period of time. In 1986, Europe established the European Working Group for *Legionella* Infections (EWGLI), which promoted the standardization of guidelines for *Legionella* disease prevention and control in member countries and travel-related settings (55). In 2010, the EWGLI was merged into the European Center for Disease Control and Prevention (ECDC). The primary goal of the current European Legionnaires' disease surveillance network is to detect, control, and prevent the dispersion, occurrence, clustering, and outbreak of the disease within the EU or European Economic Area members, with all data being shared fully with member countries (56). In 2011 and 2012, ECDC issued the Travel-Associated Legionnaires' Disease (TALD) survey, Control and Prevention Technical Guidelines, and the European *Legionella* Surveillance Network Operation Manual, providing guidelines on the diagnosis and reporting of Legionnaires' disease, in addition to the tracing and environmental treatment of cases (57, 58). In 2014, the EU revised the Water Framework Directive (WFD), which required member states to monitor and manage water in public places.^1^ With an increase in the occurrences of Legionnaires' disease in recent years, the EU has integrated the regulation of *Legionella* bacteria into EU Directive 2020/2184. The directive includes the assessment of *Legionella* concentrations among the parameters to be evaluated when determining the quality of drinking water intended for human consumption. The directive has also been extended to monitoring of *Legionella* bacteria in all drinking water distribution systems, including ample domestic water and industrial distribution systems. The limit for *Legionella* species was established at 1,000 CFU/L. Notably, the EU directive requires testing for *Legionella* spp. and not just *L. pneumophila*.^2^

### 5.3 United States

Legionellosis is required to be reported to the US Centers for Disease Control and Prevention (CDC) as a nationally notifiable disease, monitored using both the National Notifiable Diseases Surveillance System (NNDSS) and Supplemental Legionnaires Disease Surveillance System (SLDSS).^3^ (59). *Legionella* regulations were adopted in the U.S. immediately after the 1976 *Legionella* outbreak in Philadelphia (40). The U.S. Environmental Protection Agency (EPA) revised the Safe Drinking Water Act (SDWA) in 1986, explicitly adding *Legionella*-related regulations for public water systems and specifying that the number of *Legionella* detected in residential drinking water should be zero (60). The 2021 American Society of Heating, Refrigerating and Air-Conditioning Engineers (ASHRAE) Standard 188 establishes the minimum number of *Legionella* bacteria in building water supply systems.^4^ In 2021, the CDC published the *Legionella* Control Toolkit, which provides control measures for familiar sources of *Legionella* as well as recommendations for the design and testing of methods for *Legionella*, and recommendations for new methods in the future (61). In 2024, the U.S. CDC released a CDC Yellow Book Legionnaires' Disease & Pontiac Fever, providing an overview of the current research on *Legionella* bacteria (11).

### 5.4 Japan

In 1993, Japan adopted an amendment to the Drinking Water Sanitation Law that required water treatment facilities to control *Legionella* in drinking water (62). The Ministry of Health, Labour and Welfare has issued a series of guidelines for the prevention and control of *Legionella* infections, such as the “Guidelines for Water Quality Standards, for Public Baths,” which stipulates that the abundance of *Legionella* bacteria in bathing water must be <10 CFU/100 mL, and the “New Version of the Outline of Prevention of *Legionella* Bacteria,” which stipulates that when *Legionella* bacteria counts of 2 CFU/mL or more are found in non-inhalable water for human beings, disinfecting measures should be taken until the detected bacterial counts are <10 CFU/100 mL. *Legionella* counts in human inhalable water (e.g., bathtub water and shower water) should be <10 CFU/100 mL. If *Legionella* is detected, measures such as cleaning and disinfection should be taken immediately.

### 5.5 Australia

Australia has not established a dedicated monitoring system for *Legionella;* however, as *Legionella* is a nationally recognized contagious disease in Australia, all cases need to be reported to NNDSS, and infectious disease outbreaks are reported on a quarterly basis. The relevant standards for controlling and preventing *Legionella* in cooling systems include the Australian/New Zealand standard AS/NZS 3666: 2011, “Air Treatment of Buildings and Microbial Control in Water Systems,” which specifies the minimum requirements for operating and maintaining air and water supply systems in buildings for microbial control purposes (63). Further guidance can be found in other relevant Australian and Australian/New Zealand Standards and State and Territory guidelines (64). In 2016, the Environmental Health Standing Committee (enHealth) released Guidelines for *Legionella* control, which assists facility managers to assess the risk from *Legionella* in health and aged care facilities (65).

### 5.6 Poland

In Polish law, Legionellosis has been classified as an infectious disease and is regulated in the revised bill on the prevention and control of human infections and infectious diseases in 2008. In March 2007, in the revised law on drinking water quality, “the Minister of Health Regulations” pointed out that four points in the hot water system need to be tested for the presence of *Legionella*. The regulatory annex specifies the microbiological requirements that hot water should meet, and the number of *Legionella* colony forming units in 100 ml of hot water should not exceed 100. However, in closed medical facilities for patients with immunodeficiency (including those receiving immunosuppressive treatment), a 1,000 ml of water sample should not contain *Legionella*. The revised Regulations of “the Minister of Infrastructure”, revised on March 12, 2009, stipulate the technical conditions that buildings should meet, and require the maintenance of hot water temperatures within the 55–60°C during network construction (66). In recent years, due to *Legionella* outbreak in Poland, *Legionella* management has been of great concern, and the country lacks national guidelines for risk management. Sporadic outbreaks of *Legionella* have been reported before, and between 2017 and 2021, Poland reported 38 and 74 cases of Legionnaires' disease annually to the European Surveillance System (TESSy) (67).

### 5.7 India

Water safety regulations for *Legionella* monitoring and decontamination are absent in India, and there have been sporadic reports of *Legionella* in specific locations. However, so far, no outbreaks of the disease have been detected. As a developing country with limited resources, more research is required to understand the disease burden caused by *Legionell*a to improve *Legionella* testing accuracy (68, 69).

### 5.8 China

China has a different approach to the monitoring and managing Legionellosis than other countries, such as the United States and Europe; there is no monitoring system for Legionellosis, and it has not yet been classified as a legally reportable infectious disease. In 2003, the Ministry of Health promulgated the “Hygienic specification of central air conditioning ventilation system in public places” (WS 394-2012), which is an essential basis for the prevention and treatment of *Legionella* in China. The Norms stipulate that *Legionella* bacteria should not be detected in centralized air-conditioning cooling water and condensate. In 2023, the Ministry of Health formulated “Measures for the hygienic management of concentrated air conditioning ventilation system in Public Places”, “Hygienic specification of central air conditioning ventilation system in public places” (WS 10013–2023), “Hygienic evaluation specification of central air conditioning ventilation system in public places” (WS/T 10004–2023), and “Specification of cleaning and disinfecting for central air conditioning ventilation system in public places” (WS/T 10005–2023).^5^^−^^7^ These standards were officially implemented on May 1, 2024 to clarify the monitoring and prevention of *Legionella* infections.

Beijing took the lead in 2007 by introducing the local standard “Hygienic Management Specification for Central Air Conditioning and Ventilation Systems in Public Places” (DB11/485-2007),^8^ providing norms and guidance for the cleaning and disinfection of central air conditioning and ventilation systems throughout the city. In subsequent revisions, public and residential buildings were included under the jurisdiction, gradually incorporating *Legionella* detection methods for central air conditioning and ventilation systems to provide a basis for effective *Legionella* prevention. In 2016, Beijing established a Legionnaires' disease early warning technology for cooling towers in public buildings, comprehensively analyzing the reproduction, transmission, and population infection risks of cooling towers from the perspective of airborne diseases, expressing their risk levels intuitively with an index that is of great significance for the early warning and prevention of Legionnaires' disease outbreaks in public buildings (70). This technology has been piloted in the Beijing Public Health Hazard Factor Active Monitoring System since 2016, received project funding for promotion in 2017, and its promotion and application in the disease control centers of 16 districts in Beijing was completed in 2018, being officially included in the city's Public Health Hazard Factor Active Monitoring System. In 2020, the focus of the technology in the annual health and population health status report of Beijing was on conducting risk classification assessments for cooling towers in theaters, hospitals, offices, shopping places, or accommodation places.^9^ Owing to its high timeliness, the technology has been widely utilized to ensure various large-scale events. Hong Kong has issued the “Guidelines for the Prevention of Legionnaires' Disease” and the “Guidelines for Cleaning Drinking Water Tanks.” In contrast, the Macau Water Supply Department issued Decrees No. 2 and No. 33, stipulating control indicators and risk control measures for *Legionella* in cooling water systems and domestic water.

Currently, no vaccine is available for the prevention of Legionnaire's disease. Prevention measures mainly focus on controlling and regularly monitoring water quality. Mandatory measurement indicators related to facility hygiene conditions are utilized to minimize or avoid conditions for bacterial reproduction and survival, such as biofilm formation, including maintenance, cleaning, and disinfection, and to ensure the regular operation of water facilities and related devices to reduce or prevent the spread of aerosols.

## 6 *Legionella* monitoring technology

To better monitor and prevent the spread of Legionnaires' disease, relevant personnel have conducted in-depth research on environmental monitoring of *Legionella*. Collecting and testing samples from water sources and air enable measures to be taken against infection sources, and accurate and efficient detection of *Legionella* is crucial for its prevention and control.

### 6.1 Sampling techniques

Water sampling typically refers to collecting samples from water sources. This can be done by sampling contaminated building environments, such as hot water pipes or cooling towers, to help identify the source of infection and infection risks. Water sampling needs to be adjusted according to the specific environment. The detection of *Legionella* is easier in sampling water that has been stagnant for a long time, such as cooling towers or shower water. Collins et al. (71) collected water samples from household showerheads, with the showerhead being unused for several hours before sampling. The water temperature was set to the usual temperature, and the first liter of water was collected in a sterile bottle containing sodium thiosulfate. *Legionella* was detected in 8% of the water samples.

Aerosol sampling equipment should have a high collection efficiency over a wide range of particle sizes to avoid underestimating the infection risk of airborne transmission. Additionally, structural and biological damage may occur during the sampling process, which could lead to inappropriate results in subsequent quantitative analyses. The Coriolis cyclonic sampler can effectively collect *Legionella* spp. Nocker et al. (72) used a Coriolis sampler to collect exhaust gas samples below the exhaust plane of a cooling tower or above the water distribution level of a naturally ventilated cooling tower (system C) in the upwind direction, with only the aerosol detection of cooling tower system B being positive. Niculita-Hirzel et al. (73) used a Coriolis air sampler to sample *Legionella* aerosols from two types of showerheads that emitted similar levels of biological aerosols, accounting for only 0.02% of the total bacteria in the water. Montagna et al. (74) collected *Legionella* samples from medical facilities using a Coriolis sampler. The collection efficiency of the Coriolis sampler does not exceed 50%, and the bacterial concentration often falls below the actual results. Therefore, future considerations should include calibration or correction factors.

### 6.2 Detection techniques

There are multiple methods of detection and quantification of *Legionella*, and we need to better understand their limitations and the relationships among them. The International Organization for Standardization (ISO) provides two options for the detection and quantitation of *Legionella* contamination in water systems. ISO11731: 2017-05 is a culture-based method for detecting only culturable *Legionella* (75), and it is considered the gold standard. However, it is time-consuming and takes 10–14 days, but displays errors in detecting viable but non-culturable (VBNC) *Legionella*, resulting in underestimated and false negatives of *Legionella* numbers (76). Chen et al. (77) and Pan et al. (78) used culture isolations to detect *Legionella*. However, this method has a sensitivity ranging from 50 to 70%, has a long detection period, and is not suitable for rapid testing in emergencies. In addition, ISO/TS12869:2019 is a quantitative PCR (qPCR) based assay, which estimates bacterial genomic load (79). In contrast, the qPCR method is more rapid; however, it quantifies live, VBNC and dead *Legionella*, resulting in overestimations and false positive. Sánchez-Parra et al. (80) employed a dual PCR detection method targeting the V3 and V5 hypervariable regions of the 16S rRNA gene to precisely to identify pathogenic *L. pneumophila* in low-concentration outdoor DNA samples precisely.

Colloidal gold immunochromatography is commonly used for early rapid diagnosis in on-site monitoring to prevent outbreaks and the spread of *Legionella*. Ren et al. (81) prepared colloidal gold immunochromatographic test strips for rapid *Legionella* detection and conducted performance tests. The test strips did not cross-react with other pathogens, enabling fast detection; however, they may not be suitable for environments requiring high accuracy. Strain-typing methods can differentiate between different isolates of the same bacterial species and determine the cause of an outbreak. Currently, DNA sequence-based typing (SBT) serves as the reference standard for strain typing *L. pneumophila* isolates (82). Due to a disproportionate representation of some sequence types among the over 2,000 types in clinical cases, SBT may neither differentiate between outbreak-related and non-outbreak-related *Legionella* isolates nor assess changes over time within sequence types (STs) (83). Whole genome sequencing (WGS) is an essential tool for elucidating the epidemiology of these pathogens. WGS has been used in real-time to determine the sources of *Legionella* in individual and outbreak patients and to study the epidemiology of *Legionella* in facilities over time (84–86).

Currently, efforts are being made to improve *Legionella* detection, particularly in the identification of VBNC bacteria that may pose a risk to in clinical setting. As the ISO sample processing protocol has been adopted for detecting and quantifing VBNC *Legionella*, the number of VBNC *Legionella* observed in mixed bacterial cultures has increased significantly (87). Furthermore, a Chip-based duplex real-time PCR has been developed for water quality monitoring that can detect VBNC *Legionella* (88). Innovative techniques such as the PVT VIABLE™ method, have also been introduced for detection of *Legionella* that cannot form colonies using the traditional spread plate method (such as those in the ISO protocol) (89). Exposure to environmental stress and disinfection treatments promotes the formation of resistant and potentially infectious VBNC *Legionella*, and the presence of VBNC *Legionella* hinders the management of engineered water systems to prevent *Legionella* disease (87, 90). Advancing *Legionella* detection methods could improve our understanding of the role that VBNC cells play in the spread of Legionellosis and is critical to improving the control and risk management strategies for Legionnaires' disease prevention.

## 7 Summary and outlook

The COVID-19 outbreak has brought to the forefront the issue of indoor airborne pathogens into the spotlight, with the level of risk of aerosolized pathogens being exceptionally high in enclosed buildings. Increasing urbanization has led to increased population densities and the widespread use of modern equipment, which increases the exposure of the population to *Legionella* and leads to a continued rise in disease incidences. *Legionella* is transmitted from various sources and can be spread over long distances via aerosols. The possibility of aerosols being discharged from outdoor sources, transported over long distances, and entering the built environment is thus realistic, especially considering the efficient penetration of outdoor bioaerosols into the indoor environment (91). Despite significant progress in our understanding of the sources and spread of *Legionella* in the built environment, many issues are remain overlooked.

This review provides insights into *Legionella* transmission risk in the built environments for improved surveillance of *Legionella* in such environments, targeted development of preventive control strategies, and further systematic and exploratory research. There is a wide range of sources of *Legionella* in the built environment, and further research is necessary to determine whether neglected sources pose a risk of disease transmission. *Legionella* regulations and standards in developed countries have been established early and include well-established surveillance systems. However, in developing countries, such as China, where Legionnaires' disease has not yet been listed as a national statutory infectious disease, there is a deficiency in surveillance and a need to raise awareness of the severity of the problem, as effective regulations can reduce risk of transmission. Multiple approaches are available to detect and quantify *Legionella*. Non-culture-based methods can complement culture-based methods, and WGS development has paved the way for an improved analysis of the sources of *Legionella* infections and the emergence of new causative organisms, which are still relatively unexplored. Moreover, there is considerable uncertainty concerning quantitative microbiological risk assessment of *Legionella* infections. Frameworks should prioritize appropriate aerosol exposure modeling approaches, consider trade-offs between model validity and complexity, and incorporate research that strengthens confidence in quantitative microbial risk assessment (QMRA) results.

Green buildings have exacerbated many of the problems with *Legionella* species by extending the duration of water residence (leading to the loss of disinfectant residues) and lowering the hot water temperature in the house pipe. While vigorously promoting “green” building functions, focus should be directed at such emerging problems and more forward-looking *Legionella* control strategies. The certification standards for green building, energy saving functions, and water saving functions should be adapted to account for the risk factors for the growth of *Legionella* and other water-based pathogens in the building water supply system. While protecting public health, significant water conservation can still be achieved through more open water age management, for example, by regularly flushing the water to be consumed. In conclusion, the persistence of *Legionella* in green or zero-energy buildings is a complex problem requiring a multidisciplinary approach to address the challenges posed by energy conservation, water saving, and microbial contamination. Efforts to balance such factors while ensuring the safety and health of building users are essential for the design and maintenance of sustainable buildings.

The contribution of climate change to the spread of infectious diseases such as *Legionella* is increasingly being recognized. Advanced studies have been conducted to longitudinally examine Legionellosis case data about hydrometeorological factors to understand the impact of weather and climate on increased morbidity (92–96). China joined the Paris Climate Change Agreement in 2016, and research on climate change and health-related topics has been expanding. However, there is still a gap in research related to Legionellosis. Future research should also further investigate the exposure pathways between changes in hydrometeorological factors and *Legionella* infections in the built environment, and find simple interventions around water-to-air transmission to reduce the spread of *Legionella* and protect populations from infection. An in-depth understanding of the risk of Legionellosis transmission in buildings could provide the theoretical basis, technical methods, and practical applications for the prevention and control of the next airborne disease pandemic.

## Author contributions

XHY: Resources, Conceptualization, Writing – review & editing, Writing – original draft. FS: Writing – review & editing, Supervision, Resources, Project administration, Funding acquisition, Conceptualization. JH: Writing – review & editing, Resources, Investigation. LH: Writing – review & editing, Resources, Investigation. BK: Writing – review & editing, Resources, Investigation.

## Footnotes

^1^Commission Directive 2014/101/EU of 30 October 2014 amending Directive 2000/60/EC of the European Parliament and of the Council Establishing a Framework for Community Action in the Field of Water Policy Text with EEA Relevance. (2014). Available online at: https://eur-lex.europa.eu/eli/dir/2014/101/oj.

^2^European Parliament and of the Council. Directive (EU) 2020/2184 of the European Parliament and of the Council of 16 December 2020 on the Quality of Water Intended for Human Consumption. Official Journal of the European Union (2020). p. 435.

^3^US CDC (2012-12-07). *National Notifiable Diseases Surveillance Saystem (EB/OL)*. Available online at: http://wwwn.cdc.gov/nndss.

^4^ANSI-ASHRAE 188-2018. *Legionellosis: Risk Management for Building Water Systems*.

^5^WS 10013–2023. *Hygienic Specification of Central Air Conditioning Ventilation System in Public Places*.

^7^WS/T 10005–2023. *Specification of Cleaning and Disinfecting for Central Air Conditioning Ventilation System in Public Places*.

^8^DB11/485-2007. *Hygienic Management Specification for Central Air Conditioning and Ventilation Systems in Public Places*.

^9^Beijing Municipal Government. *Annual Report on Health and Population Health in Beijing. People's Medical Publishing House*.
